# Action Video Game Experience Related to Altered Large-Scale White Matter Networks

**DOI:** 10.1155/2017/7543686

**Published:** 2017-06-15

**Authors:** Diankun Gong, Weiyi Ma, Jinnan Gong, Hui He, Li Dong, Dan Zhang, Jianfu Li, Cheng Luo, Dezhong Yao

**Affiliations:** ^1^Key Laboratory for NeuroInformation of Ministry of Education, School of Life Science and Technology, University of Electronic Science and Technology of China, Chengdu 610054, China; ^2^Center for Information in Medicine, University of Electronic Science and Technology of China, Chengdu 610054, China; ^3^School of Human Environmental Sciences, University of Arkansas, Fayetteville, AR 72701, USA

## Abstract

With action video games (AVGs) becoming increasingly popular worldwide, the cognitive benefits of AVG experience have attracted continuous research attention over the past two decades. Research has repeatedly shown that AVG experience can causally enhance cognitive ability and is related to neural plasticity in gray matter and functional networks in the brain. However, the relation between AVG experience and the plasticity of white matter (WM) network still remains unclear. WM network modulates the distribution of action potentials, coordinating the communication between brain regions and acting as the framework of neural networks. And various types of cognitive deficits are usually accompanied by impairments of WM networks. Thus, understanding this relation is essential in assessing the influence of AVG experience on neural plasticity and using AVG experience as an interventional tool for impairments of WM networks. Using graph theory, this study analyzed WM networks in AVG experts and amateurs. Results showed that AVG experience is related to altered WM networks in prefrontal networks, limbic system, and sensorimotor networks, which are related to cognitive control and sensorimotor functions. These results shed new light on the influence of AVG experience on the plasticity of WM networks and suggested the clinical applicability of AVG experience.

## 1. Introduction

Originally designed for entertainment purposes, action video games (AVGs) have also attracted increasing research attention, as they offer a unique perspective experience-related brain plasticity [1]. This may be due to the fact that AVG playing requires cognitive abilities [2] in a manner similar to conventional sports (e.g., basketball).

According to the behavioral evidence over the past two decades, AVG experience can causally improve cognitive control and sensorimotor abilities. For example, AVG experience can enhance selective attention [3], spatial distribution of visuospatial attention [4], and attentional capture [5]. Furthermore, AVG experience can improve the behavioral performance of tasks in working memory [6], vision [7], multisensory temporal processing abilities [8], eye-hand motor coordination [9], and response speed [10].

Although little, neuroscience evidence also supports the cognitive benefits of AVG experience. For example, Granek et al. showed the cortical network features of extensive AVG experience related to enhanced visuomotor transformation [11]. AVG experience is also related to increased gray matter volume (GMV) in dorsal striatum [12]; right posterior parietal [13], entorhinal, hippocampal, and occipital volume [14]; and dorsolateral prefrontal cortex [15], which are related to improved performance on cognitive control and sensorimotor functions. Our recent study also found that AVG experience was related to increased resting state functional connectivity (rsFC), mainly involving attentional and sensorimotor networks [16].

However, little research has examined the relation between AVG experience and the plasticity of white matter (WM) fiber networks. Understanding this relation is essential in assessing the influence of AVG experience on neural plasticity. First, WM network modulates the distribution of action potentials, coordinating the communication between brain regions and acting as the framework of neural networks [17]. Thus, WM network offers an important venue to examine experience-related plasticity of neural networks. Second, various types of cognitive deficits are usually accompanied by impairments of WM networks. For example, the evidence showed that patients with attention-deficit hyperactivity disorder (ADHD) have impaired attentional WM network [18, 19]. Thus, understanding this relation will help to use AVG experience as an interventional tool for impairments of WM networks.

Using *diffusion tensor image* (DTI, a noninvasive technique tracking WM fibers of brain) and *graph theory* analysis, this study examined WM networks in AVG experts and amateurs in terms of global and nodal characteristics and structural connections. Graph theory is the study of graphs, which are mathematical structures used to model pairwise relations between objects. A graph is a set of nodes (vertices) linked by connections (edges) and provides an abstract representation of the elements and their interactions in a system. Graph theory has been widely used to quantitatively characterize topological organization of neural networks [20].

Specifically, we first examined WM network with global characteristics in terms of global efficiency, mean clustering coefficient, and local efficiency. Then, we further examined local characteristics of WM network in terms of connections, nodal efficiency, nodal strength, and nodal clustering coefficient. Global characteristics were used to investigate potential alterations of WM networks in a whole brain level. And local characteristics were used to investigate in detail the alteration of global characteristics in a local brain area level. To control confounding variables, two groups were carefully matched based on demographic and behavioral data. We predict that if AVG experience can enhance cognitive functions, experts should have altered WM networks compared to amateurs.

## 2. Materials and Methods

### 2.1. Participants

The experimental protocols were approved by the ethics research committee of the University of Electronic Science and Technology of China (UESTC) and were performed in accordance with ethical standards outlined by the Declaration of Helsinki. Informed consents were obtained from all subjects.

Twenty-eight AVG experts, males (*M* = 24.6 ± 1.4 yrs.), and 30 amateurs, males (24.3 ± 1.8 yrs.), participated in this study. The AVG experts had at least 6 years AVG tournament and training experience and were recognized as either regional or national champions. The experts' AVG experience was quantified based on their ladder rank score, ranging from 1900 to 2600 points, measured on Elo's chess skill rating scale [21]. All the amateurs had less than 1200 points. The Elo rating scale is widely used as a rating system for multiplayer competition in AVGs. The difference in the ratings between two players serves as a predictor of the outcome of a match. A difference of 100 points indicates that the probability of winning an AVG match for the stronger player is 64% and 200 points is 76%. Confounding variables (age, educational experience, Raven's progressive matrices, academic record, and the onset age of playing AVG) were matched between groups. The only significant between-group differences were *weekly average time* (AT) spent on playing AVG. The experts' AT were correlated with their ladder rank scores (*r*_points_ = 0.53, *p* = 0.004), suggesting that AT is a sensitive indicator of AVG expertise. All the participants were right handed according to the Edinburgh Inventory [22], reported to have normal or corrected-to-normal vision and normal hearing, and presented no history of neurological illnesses.

### 2.2. Data Acquisition

Images were acquired on a 3T MRI scanner (GE Discovery MR750) at the MRI Research Center of UESTC. Anatomic 3D T1-weighted axial images were acquired using a spoiled gradient recall (SPGR) sequence that covered the entire brain (152 slices, TR = 6.008 msec, TE = 1.984 msec, matrix = 256 × 256, FOV = 25.6 cm × 20 cm, and flip angle = 90°). The DTI acquisition used a single-shot spin-echo planar imaging sequence (TR = 8, 500 msec, TE = 66.6 msec, matrix = 128 × 128, FOV = 25.6 cm × 25.6 cm, thickness = 2 mm without gaps, and 75 slices covered the whole brain). Three unweighted B0 (*b* = 0 s/mm) and 64 diffusion-weighted (*b* = 1000 s/mm) images were collected.

### 2.3. Data Preprocessing

For each participant, the 15 diffusion-weighted scans were aligned to the first unweighted B0 image (*b* = 0 s/mm) to minimize slight head movements using the SPM8 software package (Statistical Parametric Mapping, http://www.fil.ion.ucl.ac.uk/spm/software/spm8).

### 2.4. Network Node Definition

The definition of nodes was based on the procedure used in previous studies [23]. Specifically, for each participant, the T1-weighted structural image was first coregistered to its B0 image (*b* = 0 s/mm^2^) in the DTI native space, using a linear transformation. Second, the coregistered structural images in the DTI native space were registered to the ICBM-152 T1 template in the MNI space to obtain an affine transformation matrix, T, with 12 degrees of freedom, together with a series of nonlinear warps characterized by a set of 7 × 8 × 7 basis functions. Finally, the inverse transformation matrix T1 was utilized to warp the automated anatomical labeling (AAL) atlas [24] from the MNI space to the DTI native space, the same procedure as previous studies [25]. The procedure preserved discrete labeling values using the nearest-neighbor interpolation method in SPM8. After the completion of the above procedure, the cerebral cortex and subcortex for each participant were anatomically parcellated into 90 regions of interest (ROI), 45 for each hemisphere, excluding the region of the cerebellum. Each ROI represents one node of the WM network in a participant.

### 2.5. WM Fiber Tractography

The corrected diffusion-weighted images and B0 images were used to reconstruct the whole brain WM tracts for each participant. The diffusion tensor was estimated by the linear least-squares fitting method at each voxel, using the interactive software Diffusion Toolkit 0.6.2 [26], and whole brain fiber tracking was performed in the DTI native space [27]. During tracking, the fiber assignment by continuous tracking algorithm was employed [28]. If the FA value was less than 0.15 or the angle between the current and the previous path segment was higher than 35 degrees, the path tracking was stopped. After whole fiber tracking, any fiber shorter than 20 mm or longer than 300 mm and obvious false paths were discarded [29]. To ensure that each brain region was sufficiently in contact with the fibers, they were expanded 2-3 mm into the white matter. Fiber bundles connecting each pair of brain regions were extracted from the total collection of brain fibers.

### 2.6. Network Edge Definition

Two regions (regions *i* and *j*) were considered structurally connected if at least one fiber bundle with two endpoints was located in these two regions [30]. According to the number of fibers linking region *i* and region *j*, the weight of the edge linking regions *i* and *j* was normalized: *w*(*i*, *j*) = *N*_*i*,*j*_/max(*N*_*i*,*j*_), where *N*_*i*,*j*_ is the number of fibers linking regions *i* and *j*, and max(*N*_*i*,*j*_) is the maximum number of fibers linking any two nodes in graph G. After the above procedure, a weighted WM network, represented in a symmetric 90 × 90 matrix, was constructed for each participant.

### 2.7. Connectivity Backbone

Because of noise and limitations in tractography, the risk of false-positive connections exists. To limit this risk, a connectivity backbone was estimated according to the aforementioned network for each participant [30]. First, a maximum spanning tree, which connects all nodes of the network such that the sum of its weights is maximal and in which there are no cycles, was extracted. Then, additional edges were added in order of their weights until the average node degree (the degree of a node is the number of edges connected to that node in a graph) was *K*. To insure the sparseness and efficiency of the network, *K* was set as 4 according to our previous experience and a previous study [30]. All subsequent network (graph) analyses and visual representations were based on the resultant network (connectivity backbone).

### 2.8. Graph Theory Analysis

In this study, we used the Brain Connectivity Toolbox (http://www.brain-connectivity-toolbox.net) to analyze the following network and nodal characteristics. Detailed mathematical formulas are presented in Table 1.

### 2.9. Statistical Analysis

The comparison analysis at the group level was performed using a nonparametric approach (permutation test), which was usually used when the normality assumption was violated [31, 32]. For a given parameter, we first estimated the *t* value to indicate the between-group difference. Then, we randomly assigned the parameter values for all subjects in this study into two groups to recalculate the *t* value between the two randomized groups. We repeated the permutation 10,000 times and obtained 10,000 *t* values. Finally, we determined the significance level of the between-group differences at 95% of the empirical distribution in a two-tailed test, also see [33]. Partial correlations were computed between characteristics and behavior data with age controlled.

## 3. Results

### 3.1. Global Characteristics

Compared to the amateurs, AVG experts had three significantly increased global characteristics, including global efficiency, mean clustering coefficient, and local efficiency (*p* < 0.05 Bonferroni corrected).

### 3.2. Structural Connections

**Figure 1:**
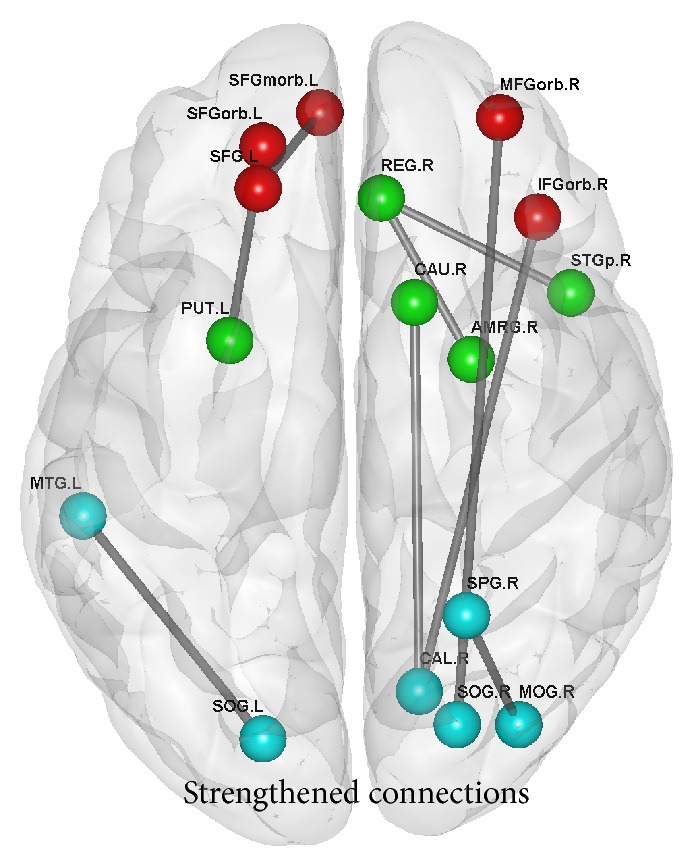
The differences in structural connection at the group level. See Table 1 for full names of nodes. Red nodes were located at the prefrontal network; green nodes were at the limbic system; blue nodes were at the sensorimotor network (L = left, R = right). The gray lines denote the structural connection where experts had significant enhancements compared with amateurs.

Compared to the amateurs, AVG experts had significantly strengthened structural connections *among* the three brain networks—the prefrontal network, the limbic system, and the sensorimotor network (*p* < 0.005, uncorrected). Furthermore, the experts did not have significantly weakened structural connections (see Figure 1).

### 3.3. Nodal Characteristics

**Figure 2:**
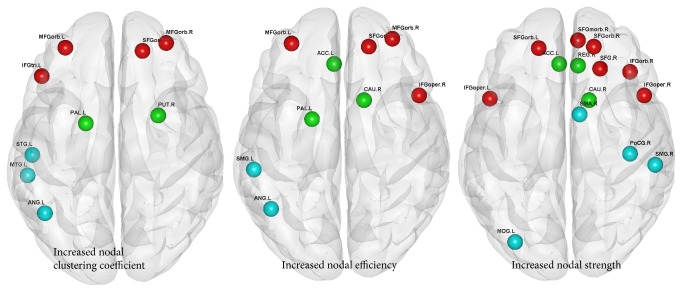
The differences in network and nodal characteristics at the group level. Red nodes were at the prefrontal network; green nodes were at the limbic system; blue nodes were at the sensorimotor network (L = left, R = right). All nodes denote the network or nodal characteristics where experts had significant enhancements compared with amateurs.

Significantly increased node characteristics were found in AVG experts compared to amateurs, including nodal clustering coefficient, nodal efficiency, and nodal strength (*p* < 0.005 uncorrected) (see Figure 2). These significant nodes had a similar spatial distribution with significant nodes of structural connection (see details in Table 2).

### 3.4. Correlational Analyses

In the expert group, we found that AT was significantly correlated with the global efficiency (*r* = 0.385, *p* = 0.048), nodal efficiency (*r*_nodal efficiency REG−AT_ = 0.395, *p* = 0.033; *r*_nodal efficiency CAU.R−AT_ = 0.41, *p* = 0.031; and *r*_nodal efficiency SFG.R−AT_ = 0.35, *p =* 0.041), and nodal clustering coefficient (*r*_CAU.R−AT_ = 0.423, *p* = 0.022), respectively.

## 4. Discussion

This study investigated the relationship between AVG experience and the plasticity of WM networks by comparing AVG experts and amateurs. The analyses on global characteristics, structural connections, and nodal characteristics showed altered WM networks in AVG experts compared to amateurs. The alterations were evident in the prefrontal network, the limbic system, and the sensorimotor network, which are mainly related to cognitive control and sensorimotor functions according to the previous studies.

### 4.1. Increased Global Characteristics

For the global characteristics, AVG experts had significantly increased global efficiency, mean clustering coefficient, and local efficiency compared to amateurs. Global characteristics often indicate the global information of a network [34]. Specifically, the increased global efficiency is usually associated with enhanced efficiency of information communication in the whole networks; the increased local efficiency was usually expressed as enhanced fault tolerance of the network when a particular node is absent (e.g., due to Alzheimer's disease) [35]; and the increased mean clustering coefficient often indicates the advanced ability of specialized information processing in most nodes [34]. These results are consistent with our recent study which found AVG experts had increased global efficiency and mean clustering coefficient in salience and central executive networks with resting-state functional data [36]. These results are also consistent with the previous behavioral results which suggested AVG experts have better performance in strong anti-interference, mass information processing, high-speed information acquisition, and quick and accurate response [37–40]. The increased network characteristics therefore suggest that AVG experts' WM networks could integrate specialized information and tolerate risk factors more efficiently than amateurs.

### 4.2. Increased Local Characteristics

According to the functional features of brain regions, the whole brain WM network would be divided to subnetworks or subsystems. We found AVG experts had significantly strengthened connections for some key subnetworks. Specifically, we found significantly strengthened connections in the prefrontal network, limbic system, and sensorimotor network, respectively (including SFGmorb.L-SFG.L, REG.R-AMYG.R REG.R-STGp.R, MTG.L-SOG.L, and SPG.R-MOG.R). We also found significantly strengthened connections between the prefrontal networks and limbic system (SFGorb.L-PUT.L), between limbic system and sensorimotor network (CAU.R-CAL.R), and between the prefrontal network and sensorimotor network (MFGorb.R-SOG.R and IFGorb.R-CAL.R) (Figure 1). For healthy people, strengthened WM connections often indicate better WM connectivity which might support more efficient information coordination and communication between brain cortexes. Thus, these strengthened connections (both the intra- and intersubnetwork) might be important reasons in supporting AVG experts' higher global efficiency.

For the nodal characteristics, AVG experts have increased nodal clustering coefficient, nodal efficiency, and nodal strength in the prefrontal network, limbic system, and sensorimotor network. Research suggested that the nodal clustering coefficient is related to the ability for specialized information processing of nodes, nodal efficiency reflects the ability of nodes to integrate specialized information from other nodes, and nodal strength is related to the importance of nodes during the information processing [34]. Thus, these findings are considered important reasons in supporting AVG experts' higher mean clustering coefficient and local efficiency.

The correlations between the global efficiency, nodal efficiency, and nodal clustering coefficient and the AT further suggested AVG experts' advanced cognitive control and sensorimotor functions might be based on these alterations of relevant WM networks. This is very possible because AVG playing requires one to process multiple objects and battlefield landforms, to constantly make, assess, and update tactical plans under time pressure, and to manipulate units by executing over 200 bimanual actions per minute by keyboard and mouse [16]. Furthermore, cognitive control of the prefrontal network is related to the adjustment of arousal and motion in the limbic system, information input, and response output at the sensorimotor network. Thus, the alterations of WM networks might be a structural basis for efficient information communication among these brain networks.

### 4.3. The Potential Clinical Applicability

This study showed the AVG experience-related alterations on WM networks, suggesting the potential to use AVG as an interventional tool for mental and neurological deficits. For example, a recent DTI study showed decreased node efficiency in SFGorb.L, SMG.L, and ANG.L and global efficiency in ADHD patients [19]. Furthermore, Alzheimer's disease (AD) patients had decreased global efficiency and node efficiency of WM networks including SFGorb.R and MFGorb.R [41], while schizophrenia showed reduced node strength (related to IFG.R, MFG.L, and SFG.L) and node clustering coefficient (related to MFG.R and MTG.L) [42]. More importantly, these impairments in ADHD, AD, and schizophrenia patients correspond to the increases in AVG experts, thus supporting the potential clinical applicability of AVG.

### 4.4. Limitations and Interpretation

In this study, the correlational nature of this study precludes causal inferences. For example, AVG experts may have an innate advanced attentional ability, which in turn may reinforce their interest in AVG. In addition, AVG experts may lead a more active life than amateurs, which may also contribute to the structural network change. However, the logistic difficulty of retaining subjects throughout a training study often limits the duration of training, which is usually much shorter than the acquisition of expertise in the real world that may take several years. This can pose a challenge especially to the studies on the plasticity of WM, which usually occurs through long-term continuous training. Thus, research on structural WM plasticity often use a cross-sectional approach by comparing experts and amateurs [43, 44], based on the logic that if learning of specific skills does induce changes of particular brain areas, such changes should be most easily observable by comparing experts and amateurs.

## 5. Conclusions

Using graph theory, this study analyzed WM networks in AVG experts and amateurs. Results showed that AVG experience is related to altered WM networks in prefrontal networks, limbic system, and sensorimotor networks, which are related to cognitive control and sensorimotor functions. These results shed new light on the influence of AVG experience on the plasticity of WM networks and suggested the clinical applicability of AVG experience.

## Figures and Tables

**Table 1:** Mathematical formulas used in graph theoretical analyses.

Characteristics	Mathematical formulas	Interpretations
Global efficiency	$E_{global}=\frac{1}{N\left(N-1\right)}\underset{i\neq j\in G}{\sum }\frac{1}{L_{ij}}$	*E*_global_ reflects how efficiently information can be communicated over the whole network.
Local efficiency	$E_{local}=\frac{1}{N}\underset{j\neq k\in G}{\sum }E_{global}\left(G_{i}\right)$	*E*_local_ has been used to reveal the fault tolerant capability of a network.
Mean clustering coefficient	$C_{p}=\frac{1}{N}\underset{i\in G}{\sum }C_{i}$	*C_p_* is a measure of the extent of the mean interconnectivity or cliquishness in a graph.
Nodal clustering coefficient	$C_{i}=\frac{1}{k_{i}\left(k_{i}-1\right)}\underset{\begin{array}{c} j,k\in G\\ j,k\neq i \end{array}}{\sum }{\left(w_{ij}\cdot w_{jk}\cdot w_{ki}\right)}^{1/3}$	*C_i_* is a measure of the extent of the local interconnectivity or cliquishness in a graph (the edge weight (*w*)).
Nodal efficiency	$E_{i}=\frac{1}{\left(N-1\right)}\underset{i\neq j\in G}{\sum }\frac{1}{L_{ij}}$	*E_i_* depicts the importance of node *i* during the information communication within a network.
Nodal strength	$S_{i}=\underset{i\in G}{\sum }w_{ij}$	The larger *S_i_* is, the more important node *i* becomes.

We defined the subgraph *G*_*i*_ as the set of nodes that is the direct neighbors of the *i*th node, that is, directly connected to the *i*th node with an edge. The degree of each node, *K*_*i*,*i*=1,2,...,23_, is defined as the number of nodes in the subgraph *G*_*i*_.

**Table 2:** Detailed information on significant nodes. BA: Brodmann areas.

Abbr.	Full name	BA	Involved networks	Involved functions
SFGmorb	Superior frontal gyrus, medial orbital	BA 10	Prefrontal networks	Cognitive control
SFG	Superior frontal gyrus	BA 9	Prefrontal networks	Cognitive control
SFGorb	Superior frontal gyrus, orbital	BA 11	Prefrontal networks	Cognitive control
IFGorb	Inferior frontal gyrus, orbital	BA 47	Prefrontal networks	Cognitive control
MFGorb	Middle frontal gyrus, orbital	BA 46	Prefrontal networks	Cognitive control
IFGtri	Inferior frontal gyrus, triangular	BA 45	Prefrontal networks	Cognitive control
IFGoper	Inferior frontal gyrus, opercular	BA 44	Prefrontal networks	Cognitive control
REG	Rectus gyrus	BA 11	Limbic system	Cognitive control
ACC	Anterior cingulate gyrus	BA 24	Limbic system	Cognitive control
PUT	Lenticular nucleus, putamen	—	Limbic system	Motor learning and execution
PAL	Lenticular nucleus, pallidum	—	Limbic system	Regulating movements
CAU	Caudate nucleus	—	Limbic system	Spatial and motoric memory Directed movements
AMRG	Amygdala	—	Limbic system	Stressing response
HIP	Hippocampus	—	Limbic system	Spatial memory and navigation
STGp	Superior temporal gyrus, temporal pole	BA 38	Limbic system	Limbic associational integration
SMA	Supplementary motor network	BA 6	Sensorimotor network	The control of movement
MTG	Middle temporal gyrus	BA 21	Sensorimotor network	Temporal associational integration
STG	Superior temporal gyrus	BA 48	Sensorimotor network	Audio-visual integration, and motion perception
SPG	Superior parietal gyrus	BA 7	Sensorimotor network	Spatial orientation
SOG	Superior occipital gyrus	BA 19	Sensorimotor network	Processing visual information
ANG	Angular gyrus	BA 39	Sensorimotor network	Spatial orientation
CAL	Calcarine fissure and surrounding cortex	BA 18	Sensorimotor network	Processing visual information
MOG	Middle occipital gyrus	BA 19	Sensorimotor network	Processing visual information
PoCG	Postcentral gyrus	BA 3	Sensorimotor network	Processing somatosensory information
SMG	Supramarginal gyrus	BA 40	Sensorimotor network	Spatial orientation
